# Impact of self-assembling peptides in remineralisation of artificial early enamel lesions adjacent to orthodontic brackets

**DOI:** 10.1038/s41598-020-72185-2

**Published:** 2020-09-15

**Authors:** Anahita Jablonski-Momeni, R. Nothelfer, M. Morawietz, A. Kiesow, H. Korbmacher-Steiner

**Affiliations:** 1 Department of Orthodontics, Dental School, Philipps University of Marburg, Georg-Voigt-Str. 3, 35039 Marburg, Germany; 2 Fraunhofer Institute for Microstructure of Materials and Systems IMWS, Halle (Saale), Germany

**Keywords:** Diseases, Health care, Optics and photonics

## Abstract

Enamel demineralisation can occur as a side effect during orthodontic treatment with fixed appliances. This study aimed to evaluate the efficacy of the self-assembling peptide P_11_-4 for remineralisation combined with fluorides, compared to application of fluoride varnish alone. De- and remineralisation was assessed by Quantitative light-induced fluorescence (QLF). Orthodontic brackets were bonded on 108 human enamel samples and white spot lesions were created. The samples were allocated randomly into three groups: Group I received no treatment, group II had a single application of fluoride varnish (22,600 ppm), and group III was treated with P_11_-4 following a single application of fluoride varnish. Quantitative light-induced fluorescence (QLF) measurements were performed at baseline, after demineralisation and after storage in remineralisation solution for 7 and 30 days. Non-parametric tests (Kruskal–Wallis test and Friedman test) were used for further analysis. After demineralisation, all samples showed a median ΔF -9.38% ± 2.79. After 30 days median ΔF values were as followed: group I = -9.04% ± 2.51, group II = -7.89 ± 2.07, group III = -6.08% ± 2.79). The median ΔF values differed significantly between all groups at all investigation times (p < 0.00001). Application of P_11_-4 with fluoride varnish was superior to the use of fluorides alone for remineralisation of enamel adjacent to brackets.

## Introduction

During fixed orthodontic treatment demineralisation may occur adjacent to brackets. Without special prevention concepts, early enamel changes may progress and white spot lesions occur. A meta-analysis showed that the incidence of new carious lesions formed during orthodontic treatment in patients was 45.8%^1^. According to some authors^2^ high treatment demand and occurrence of biofilm-related complications make orthodontic treatment a potential public health threat.

It is well accepted that caries progression is initiated by an imbalance of the remineralisation–demineralisation equilibrium favoring demineralisation^3^. Patients with fixed orthodontic treatment are usually subject to strict caries preventive care, including application of remineralisation agents containing fluoride^4^. Together with pit and fissure sealants^5^ and parent’s motivation^6^ the application of fluoride is the oldest aid for caries prevention^7^. It was reported in a recent meta-analysis^8^ that active patient reminders and flat surface sealants or fluoride varnish around orthodontic brackets might be associated with reduced white-spot-lesion burden, but the authors conclude that further research is needed. As another novel approach the use of bioactive glass (Biomin F paste) was shown to be an effective method for enamel remineralisation^9^.

Although the use of fluorides has been shown to prevent dental caries effectively^10,11^, limitations exist for the application of fluoride when the caries lesion has already progressed to the clinically visible white spot lesion and the best possible outcome would be an arrest of the lesion’s activity^12^. It was shown that a one-time application of fluoride varnish at the beginning of an orthodontic treatment did not provide any additional preventive benefit over sufficient dental hygiene with fluoride toothpaste regarding formation of white spot lesions and gingivitis in patients who were at low to moderate caries risk^4^. Nevertheless, the use of fluoride has a long history in dental practice and is accepted as a standard for caries prevention. Research is needed to evaluate new approaches which would support the effect of fluorides in preventing and arresting early caries lesions even more effectively.

Recently, biomimetic remineralisation strategies such as self-assembling peptides are considered to be a new perspective in caries prevention^13–15^. It is reported that the self-assembling peptide P_11_-4 forms a three dimensional matrix within the subsurface body of an initial enamel lesion and mimics enamel matrix proteins^16,17^. A high affinity of the P_11_-4 matrix for calcium ions is described, which acts as a nucleator for de novo hydroxyapatite formation^16–18^. Nowadays, the self-assembling peptide P_11_-4 is available in commercial products named Curolox Technology (credentis ag, Windisch, Switzerland). One available product is the CURODONT REPAIR which was developed for the in-depth biomimetic treatment of initial carious lesions.

Synthetic nanohydroxyapatite is also considered one of the most biocompatible and bioactive materials having similar morphology, structure, and crystallinity to the apatite crystal within enamel^19^. In vitro studies have shown that nanohydroxyapatite had the potential to remineralise initial enamel lesions with a comparable or even superior efficacy to that of fluoride^20,21^.

Non-operative, remineralising measures can only be used effectively if demineralisation is detected early. Beside visual, often subjective measures, the use of adjuvant procedures may provide additional benefit in objectifying and documentation of the demineralisation around orthodontic brackets and for digital demonstration. It is well known that demineralisation can be detected by light-induced fluorescence, since the fluorescence radiance at the site of a caries lesion is decreased^22^. It is possible to quantify the loss of fluorescence in a caries lesion in comparison with the fluorescence radiance level of sound enamel using systems like the Quantitative Light-induced Fluorescence (QLF). The method is suitable for detection and monitoring of mineral changes in incipient enamel lesions^23–26^.

The efficacy of P_11_-4 in remineralisation of early enamel lesions was shown using different detection methods such as laserfluorescence (DIAGNOdent)^27,28^ or impedance measurements^29^. But no published data are available using QLF as a reference standard. The aim of the present in-vitro study was to evaluate the effect of P_11_-4 in remineralisation of artificial lesions adjacent to orthodontic brackets with QLF.

## Results

A number of 108 samples were included in the study. The analysis of the QLF values after de- and remineralisation showed no significant differences between different bracket materials (Kruskal–Wallis Test, p values: after demin = 0.23, after 7d remin = 0.67, after 30d remin = 0.46). Hence, the subsequent analyses were performed between the different treatment groups without further sub-analysis according to the various bracket types.

**Figure 1:**
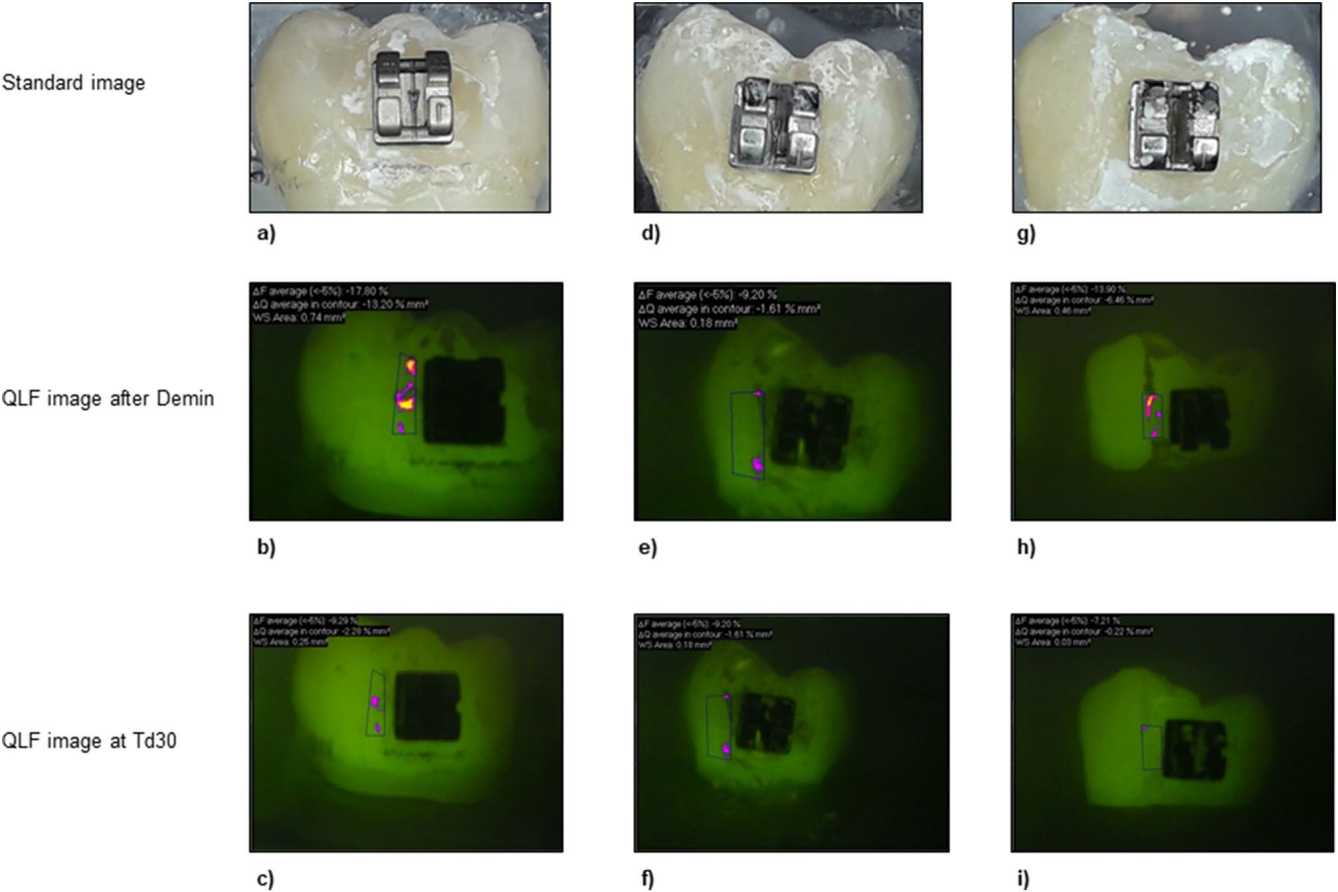
Samples of each group with the corresponding fluorescence images at T30d. Values for ΔF and ΔQ are displayed above each QLF image. **(a–c)** Sample of group I (no treatment); **(d–f)** sample of group II (single application of fluoride varnish); **(g–i)** sample of group III (application of P_11_-4 + single application of fluoride varnish).

After demineralisation, all samples showed a distinct fluorescence loss with a median ΔF of − 9.38% (± 2.79) and a median ΔQ of − 1.13% × mm^2^ (± 4.96), respectively. The values for percentage of fluorescence loss (ΔF and ΔQ) after remineralisation are summarized for each group in Tables 1 and 2. In Fig. 1, representative images of each treatment group are presented. The samples were divided equally on the different groups randomly after demineralisation.

**Table 1:** Results of the QLF measurements: ΔF (fluorescence loss [%]) in various groups (SD: standard deviation).

Time	Group	Minimum	Maximum	Mean	Median	SD
After demin	I	− 17.70	− 6.85	− 9.70	− 9.38	2.05
	II	− 18.60	− 6.90	− 10.12	− 9.43	3.13
	III	− 21.10	− 6.85	− 9.80	− 9.42	3.10
ΔF T7d	I	− 19.00	− 6.03	− 11.71	− 10.85	3.25
	II	− 16.40	− 6.37	− 10.19	− 9.73	3.24
	III	− 13.00	0.00	− 8.10	− 7.21	2.64
ΔF T30d	I	− 16.80	− 6.31	− 9.53	− 9.04	2.51
	II	− 14.40	− 6.24	− 8.29	− 7.89	2.07
	III	− 10.70	0.00	− 5.30	− 6.08	2.79

Group I = no treatment.

Group II = single application of fluoride varnish.

Group III = application of P_11_-4 + single application of fluoride varnish.

**Table 2:** Results of the QLF measurements: ΔQ (fluorescence loss [%] x area [mm^2^]) in various groups (SD: standard deviation).

Time	Group	Minimum	Maximum	Mean	Median	SD
After demin	I	− 38.40	− 0.25	− 3.29	− 1.51	6.43
	II	− 24.10	− 0.10	− 2.91	− 0.98	5.26
	III	− 12.00	− 0.01	− 1.57	− 1.15	2.10
ΔF T7d	I	− 31.50	− 0.03	− 6.45	− 3.65	6.96
	II	− 23.90	− 0.07	− 4.61	− 1.93	5.85
	III	− 15.70	0.00	− 1.46	− 0.40	3.08
ΔF T30d	I	− 13.70	− 0.05	− 3.00	− 1.13	3.86
	II	− 18.00	− 0.02	− 2.16	− 0.62	3.84
	III	− 0.31	0.00	− 0.06	− 0.03	1.17

Group I = no treatment.

Group II = single application of fluoride varnish.

Group III = application of P_11_-4 + single application of fluoride varnish.

Comparison between the treatment groups (Kruskal–Wallis test and Dunn post-hoc tests) showed the following for the fluorescence values ΔF and ΔQ: After demineralisation all values were not significantly different from each other (p values 0.925 for ΔF and 0.143 for ΔQ, respectively. This indicated an equal allocation of the samples to each treatment group after demineralisation. At T7d ΔF values in group III were significantly lower that groups I and II (p = 0.00001). The same was confirmed for ΔQ values (p = 0.000006). At T30d ΔF and ΔQ in group III were still significantly lower than in groups I and II, indicating a higher remineralisation in the test group (ΔF: p = 0.00001, ΔQ: p < 0.000001).

Multiple comparisons between different time intervals in each individual treatment group (Friedman test) showed the following results: In group I fluorescence values decreased at T7d significantly (p < 0.00001) towards demineralisation but increased at T30d towards the baseline values (Table 1 and 2). In group II the fluorescence behavior remained unchanged after 7d but the values increased at T30d significantly (p = 0.0008) indicating mineral gain after 30 days. In group III the fluorescence values increased continuously at T7d and T30d (p < 0.00001) indicating a mineral gain at each study time.

**Figure 2:**
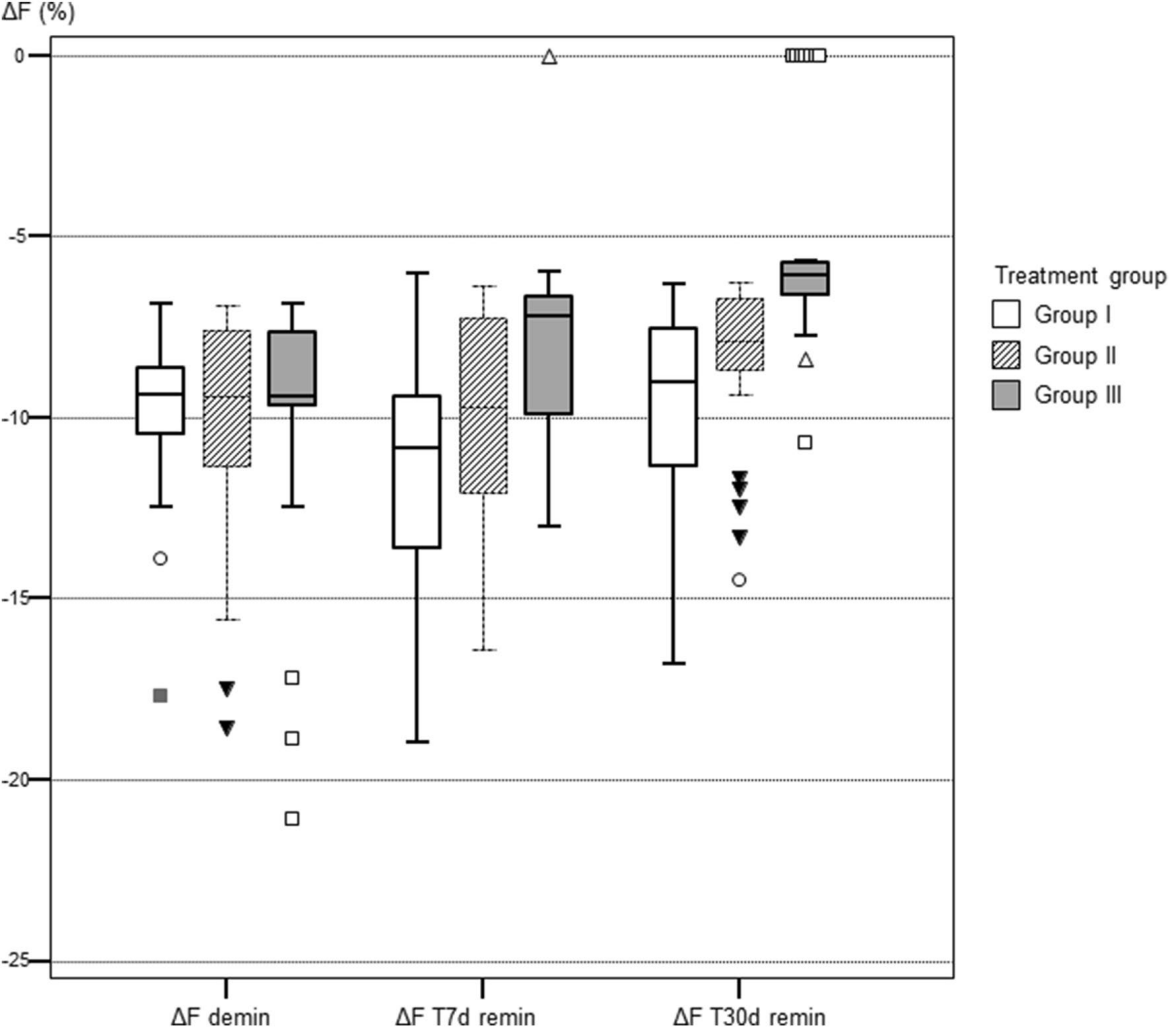
Boxplots of the fluorescence measurements (fluorescence loss ΔF in %) in each group at the different investigation times. Group I = no treatment; Group II = single application of fluoride varnish; Group III = Application of P_11_-4 + single application of fluoride varnish.

**Figure 3:**
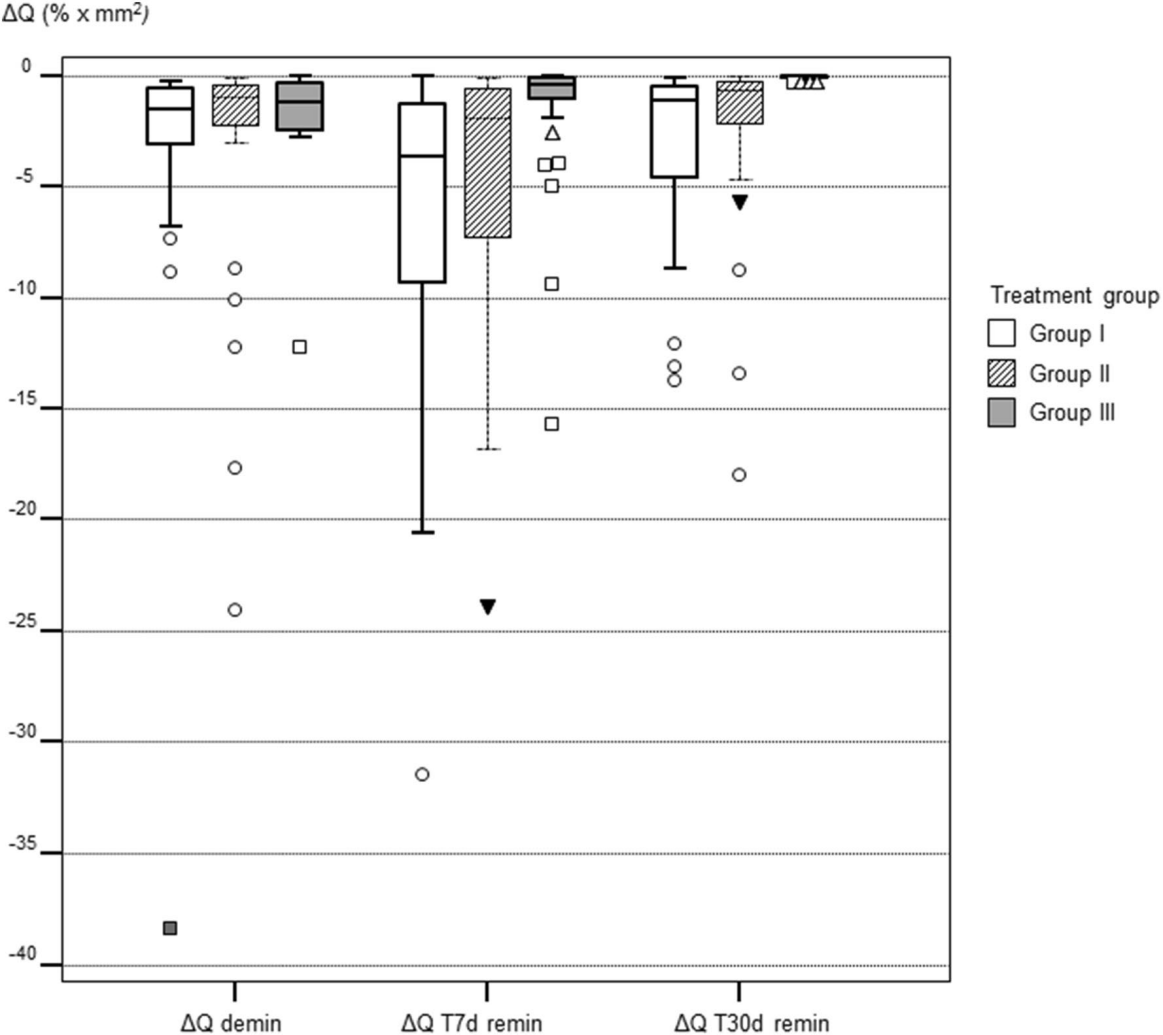
Boxplots of the fluorescence measurements (fluorescence loss times the area ΔQ in % × mm^2^) in each group at the different investigation times. Group I = no treatment; Group II = single application of fluoride varnish; Group III = Application of P_11_-4 + single application of fluoride varnish.

The corresponding box-plots are presented in Fig. 2 and 3.

## Discussion

Enamel demineralisation is known to be an undesired side effect of fixed orthodontic treatment. Initial lesions may progress towards cavities, and as a consequence orthodontic appliances may be removed prior to the proper end of the treatment^2^. Ever since, orthodontic treatment is stated to be at risk of becoming a public health threat^2^. So, it´s all the more important to modify the caries risk factors und emphasis on management strategies which focus on the remineralisation of the lesions. Such strategies are use of topical fluoride^30–32^, amorphous calcium phosphate^33^ or even a combination of both agents^34^. In addition, masking the lesions would improve the esthetic appearance of the teeth including bleaching, micro-abrasion or micro-invasive techniques such as resin infiltration^35^ with a camouflage effect. Moreover, microcrystalline hydroxyapatite (HAP) particles were suggested as suitable candidates for the prevention of demineralisation and the stimulation of remineralisation process on tooth tissue^20,36^. It was reported that after nanohydroxyapathite treatment the enamel was more mineralised^37^ and harder than demineralised enamel^38^.

In the present study the remineralisation effect of the self-assembling peptide P_11_-4 was assessed in combination with a fluoride varnish (test group), compared to the single application of the fluoride varnish and to samples which did not receive any additional treatment. In the test group significant fluorescence increase was observed after 7 and 30 days compared to the fluorescence behavior of the samples in group I and II, demonstrating a higher remineralisation in the test group (Tables 1 and 2). A study on bovine enamel samples showed that application of P_11_-4 alone did not lead to increased fluorescence using the QLF, indicating either lack of remineralisation or irregular crystals^39^. In another study on human enamel samples it could be shown that application of P_11_-4 on demineralised enamel and storage in a remineralising solution led to better surface quality than storage in distilled water^27^. The additional effect of P_11_-4 was shown in a randomized controlled trial, compared to the application of fluoride varnish alone in active occlusal initial caries lesions on erupting permanent molars^40^ where the test group showed significantly superior lesion regression. In another randomised controlled study it was shown that the size of early carious lesions on buccal surfaces was significantly reduced after application of self-assembling peptides compared to use of fluoride varnish alone^41^. The evaluation was performed visually and hence the interpretation of the size reduction was subjective. Results from a randomized in-situ study demonstrated the superior remineralising effect of the self-assembling peptide compared to fluoride use on demineralised bovine enamel^28^. The applied gel contained 1,000 ppm P_11_-4 and 900 ppm fluoride, recommended for homecare prevention. This gel should be applied twice a week after regular oral hygiene. In this context, however, the compliance of patients wearing fixed appliances with regard to their oral care routine at home should be considered and compliance-independent measures which can be applied in the dental office should be taken into account. Very recent studies could show that the application of P_11_-4 prior to bonding of orthodontic brackets doesn´t have an effect on the shear-bond strength of the brackets^42^. Hence, the additional treatment with P_11_-4 may decrease the risk of development of initial caries lesions during fixed orthodontic treatment.

Welk et al.^29^ could show the effect of P_11_-4 on orthodontic treatment induced carious lesions after the orthodontic multibracket treatment. The authors concluded that treatment of initial carious lesions with P_11_-4 led to better remineralisation of the subsurface lesions compared with the control teeth. *Doberdoli *et al*.*^15^ investigated the effectiveness of both monomeric self-assembling peptide P_11_-4 and the polymeric self-assembling peptide matrix in treatment of non-cavitated occlusal caries in children. The study showed that P_11_-4, applied either in combination with fluoride varnish or twice weekly as a gel was a superior treatment for early caries lesions, when compared to application of fluoride varnish alone.

In a meta-analysis it was indicated that a monthly use of fluoride varnish was the most effective protocol to enhance the remineralisation due to toothbrushing with a fluoride-containing dentifrice^31^. The fluorescence loss in the samples of our study indicated that the one-time application of fluoride varnish had no impact in the remineralisation process after 7 days but only after 30 days. Whereas in the test group (P_11_-4 + fluoride varnish) the fluorescence values decreased already after 7 days and a continuous significant mineral gain was still assessed at T30d. These results show clearly the additional remineralising effect of the self-assembling peptides on the short term.

As a consequence, in the clinical setting, recall intervals should be adjusted for assessing preventive interventions and the monitoring of initial lesions and reviews of behavioral and oral hygiene change plans^43^. Moreover, application of biomimetic agents can be taken into account depending on patient’s risk forward developing caries lesions. This would be a beneficial step to inhibit the progression of already established white-spot lesions adjacent to orthodontic brackets. This issue may be addressed when pH-cycling models are used to simulate oral pH fluctuation patterns and daily oral care. Moreover, the long-term effect of remineralisation agent can be better evaluated since results after pH-cycling may better be transferred to the clinical situation. This shows the limitation of the present study where a model of a certain clinical situation was considered. In our study we looked at remineralisation of already demineralised enamel and the set-up does not give any data on the inhibition of demineralisation which could be assessed using a pH-cycling model. Consequently the raised data is applicable for the remineralisation period in the mouth. Yet, as in any in vitro experiments, timelines cannot be translated to in vivo situations entirely.

The use of QLF is a common method to assess demineralisation adjacent to orthodontic brackets without sample destruction^44–48^. In QLF images the fluorescence correlates with mineral loss of a tooth surface^49^ and can be monitored over time. The ΔF corresponds to the percentage fluorescence loss with respect to the fluorescence of sound tooth tissue and is related to lesion depth, while ΔQ takes into account the area of the lesion and is related to the lesion volume^50^. The amount of fluorescence radiance loss is already validated with transversal and longitudinal microradiography and was reported to be closely correlated (r = 0.97) to the mineral loss in the lesion^49,51,52^. The method can be considered as suitable for monitoring of mineral changes in initial enamel lesions and for the evaluation of preventive measures^53^.

In the present study, different bracket materials had no significant effect on the measurements regarding de- and remineralisation. A possible deteriorating effect that fluoride containing agents can exert on orthodontic titanium wires was discussed. It was shown that the in vitro fluoride application caused an increase in friction resistance of Ni–Ti wires when compared to stainless steel wires. In vitro and in situ fluoride application caused deterioration in surface properties of Ni–Ti wires^54^. Some studies have investigated the differences between various types of brackets in terms of adhesion of cariogenic streptococci. In some studies no differences in the adherence to stainless steel, ceramic, or plastic brackets could be demonstrated^55,56^. In contrast, other studies revealed that ceramic or plastic brackets have a higher bacterial attachment rate than most of the metal brackets^57–59^. It must be considered that in the present study a demineralisation model was used which was without colonization of cariogenic microorganisms adjacent to the brackets. Therefore, this factor was not evaluated in our study. Nevertheless, outcomes of a systematic review concluded that there is currently no evidence for a possible influence of the design of the brackets (conventional or self ligating) over colony formation and adhesion of *Streptococcus mutans* and that other factors may have greater influence, such as the quality of the bracket type or the level of individual oral hygiene^60^.

### Conclusions and clinical relevance

The present study is the first which demonstrated with the QLF the effectiveness of the P_11_-4 combined with fluoride varnish adjacent to orthodontic brackets on artificially demineralised human enamel. Although there are limitations of in-vitro studies it can be concluded that application of the highly concentrated self-assembling peptide P_11_-4 prior to topical fluoridation provides additional support for the regeneration of demineralised enamel in the short- term (up to 30 days). The effectiveness in mineral gain is superior to the one-time use of fluoride varnish. The benefit is to improve remineralisation of enamel especially in high caries risk patients like those with fixed orthodontic appliances, where fluoride alone might not be sufficient.

## Material and methods

### Study preparation and selection of the extracted teeth

The study protocol and the use of extracted human teeth were approved by the Ethics Committee of the Medical Faculty of the Philipps-University of Marburg, Germany (Ref. No.: 132/19). Prior to extraction each patient was informed and consent was obtained for the use of the teeth for study purposes. The research didn´t involve human participants.

A sample size calculation was performed with MedCalc (v19.2.1) based on preliminary (unpublished) data. A number of 13 samples was calculated for each treatment group (Power 0.90, α = 0.05), assuming a difference of mean of 4 and the standard deviation of 3 in each group.

The teeth were stored after extraction for 7–10 days in a 0.5% chloramine-T bacteriostatic solution (Merck, Darmstadt, Germany)^42^. The bulk of the adherent soft tissues was carefully removed and the teeth were cleaned with rotating brush and tooth cleansing paste with RDA value of 120 (Clinpro Prophy Paste, 3M ESPE, Seefeld, Germany). The buccal surface of the teeth were examined under a stereomicroscope (Leica MS 5, Leitz, Wetzlar, Germany) at 16 × magnification to control and exclude any surface defects. The teeth were stored in distilled water for further use^27^. The teeth were cut in a mesiodistal direction and each sample was fixed on a plexiglas slide (Dia-plus, Oststeinbek, Germany).

### Preparation of the samples

On the buccal surface of each sample orthodontic brackets were bonded according to the manufacturer’s recommendation: Enamel surface was etched with 36% phosphoric acid gel for 30 s (Conditioner 36, Dentsply DeTrey, Konstanz, Germany), rinsed with water and dried using a three-in-one-syringe. Brackets were bonded in the center of each sample (Transbond XT primer and adhesive, 3M Unitek, Landsberg, Germany) and were light cured for 10 s according to manufacturer´s instruction (FlashMax P4 Ortho Pro, orthodontic light pen, CMS Dental, Copenhagen, Denmark; Peak Output Intensity: 6,000 mW/cm^2^). Different brackets were used to evaluate whether the material (stainless steel, titanium or ceramic) has an impact on the measurements. The orthodontic brackets were randomly bonded on the samples (n = 12 in each group) using the following types: equilibrium mini (material: stainless steel), equilibrium ti (material: titanium), and discovery pearl (material: ceramic composed of aluminum oxide crystals) (all brackets: DENTAURUM, Ispringen, Germany).

### Demineralisation of the samples

**Figure 4:**
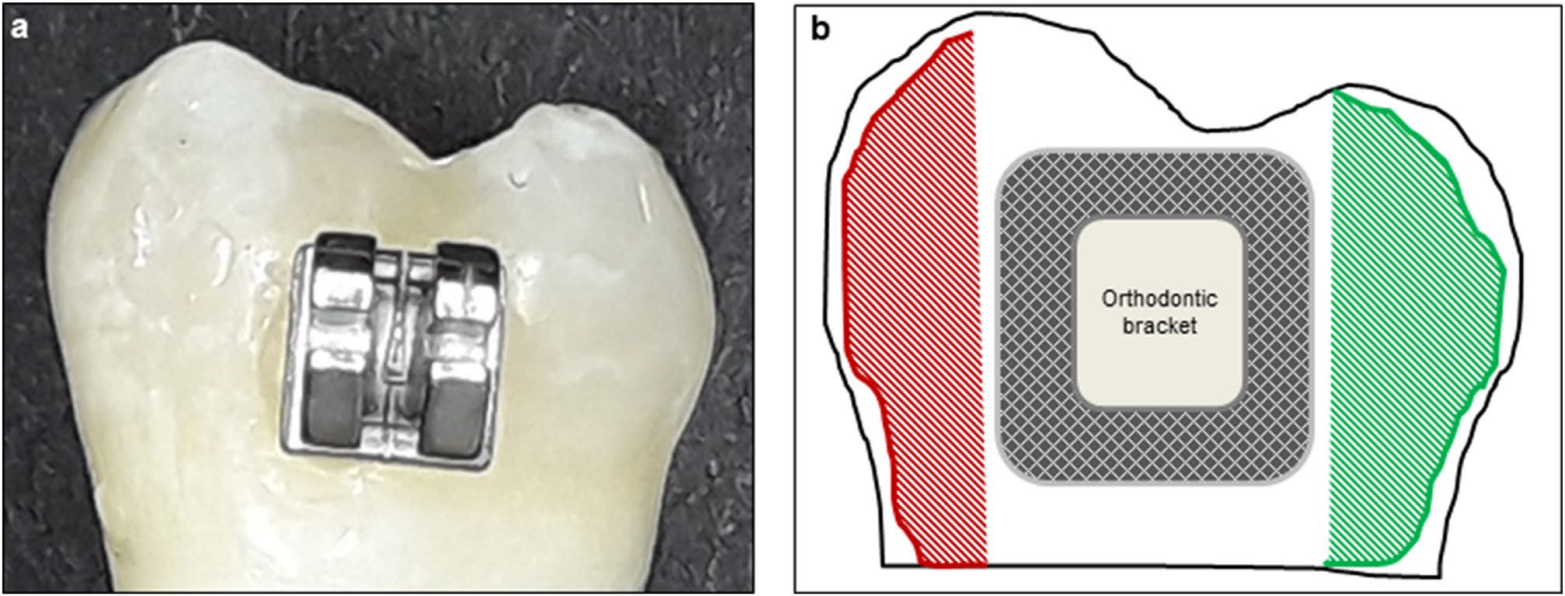
(**a)** Sample surface prior to demineralisation. (**b)** Scheme of the sample surface with different areas: Left side (red patterned area): demineralised surface. The surface was covered by nail varnish after demineralisation. Center of the surface: Orthodontic bracket. The area circular of the brackets (dark patterned area) was exposed to different remineralisation agents after demineralisation. Right side (green patterned area): sound enamel. The surface was covered by nail varnish prior to demineralisation.

A sample with different surface areas is presented in Fig. 4. On each sample, one side was covered by an acid resistance clear nail polish (Manhattan, Mainz, Germany) to protect this enamel part from demineralisation. To produce subsurface lesions, the samples were covered with a layer of a 8% methylcellulose (approximately 1.5 cm) on top of which was placed in excess 0.1 M lactate buffer, pH 4.6 ^61^, at 37** ∘**C (incubator type B, Heraeus GmbH, Hanau, Germany). After 14 days, the samples were taken out of the gel and were washed with a water jet from a three-in-one syringe to remove any gel remnants from the lesion surface. After that, the samples were rinsed with distilled water and were examined microscopically after air drying to control the surfaces for a dull, whitish surface resembling typical early enamel lesions.

**Figure 5:**
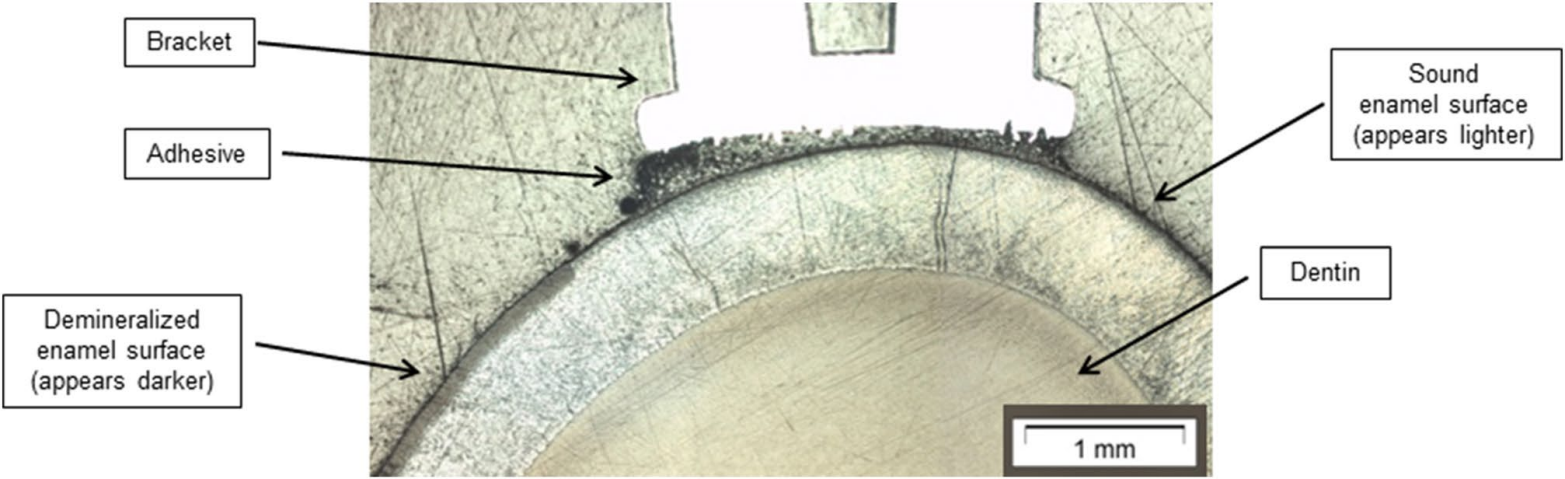
Representative cross section with the demineralised area in the enamel surface.

The depth of the demineralised enamel surface was determined on representative histological samples after sectioning. For this procedure, the samples were cut with a diamond bladed saw (Presi Minitech 333, Presi GmbH, Hagen, Germany) and embedded in epoxy resin (SpexiFix20, Struers GmbH, Willich, Germany). The cross section surface was grinded to 4,000 grit by SiC abrasive paper (SiC Struers GmbH, Willich, Germany). The sections were evaluated under a microscope (BX51, Olympus GmbH, Hamburg, Germany) for determination of lesion depth. Lesion depth was determined by image analyzer software CellF* (BX51, Olympus GmbH, Hamburg, Germany). The mean depth of the demineralised area was 97.32 µm. A representative section with the demineralised area in the enamel surface is displayed in Fig. 5.

### Assignment of the samples to the treatment groups

After demineralisation another side of the sample surface was covered by varnish in order to have a demineralised control in each sample (Fig. 4b). From each bracket group 12 samples were randomly allocated to three groups with different treatment strategies (n = 36 in each treatment group):

Group I, negative control, no treatment; group II, positive control: single application of fluoride varnish (Duraphat, containing 22,600 ppm fluoride; Colgate-Palmolive, New York, USA); group III, test group: onetime application of P_11_-4 (CURODONT REPAIR, Straumann AG, Basel, Switzerland) according to manufacturer’s instructions + single application of fluoride varnish.

The self-assembling peptide P_11_-4 was used as the clinically available CURODONT REPAIR with the chemical structure: Ace-Gln–Gln–Arg–Phe–Glu–Trp–Glu–Phe–Glu–Gln–Gln–NH2.

The material was applied to the tooth surfaces following the manufacturer´s protocol: The tooth surface was rinsed with sodium hypochlorite solution (2%, SPEIKO, Dr. Speier GmbH, Münster, Germany) to remove the pellicle. After air-drying, the surface was etched with 36% phosphoric acid gel for 20 s and was rinsed and dried thoroughly. Then the P_11_-4 was applied with its application tip. After five minutes, fluoride varnish was applied on the pre-treated surfaces. All samples were stored in a remineralisation solution, adjusted to a pH of 6.9. The solution was prepared by mixing 1.635 g KH_2_PO_4_ (2.403 mM), 1.700 g Na_2_HPO_4_ (2.395 mM), 6.335 g KCL (16.993 mM), 2.920 g NaCl (9.993 mM) and 0.830 g CaCl_2_ * 2 H_2_O (1.129 mM) in 5 L distilled water (all chemicals: Merck, Darmstadt, Germany). The pH was measured daily and the solution was replaced weekly. The samples were stored in the incubator at a constant temperature of 37 °C.

### Quantitative light-induced fluorescence (QLF)

Noninvasive measurements were performed as the reference standard for the quantification of de- and remineralisation using the Quantitative light induced fluorescence method (QLF Inspektor Pro, Inspektor Research Systems, Amsterdam, Netherlands). The QLF analysis software Inspektor Pro 2.0.0.48 was used to quantify the fluorescence behavior of the enamel surfaces which is directly proportional to the mineral content^62^. The measurements were performed at baseline, after demineralisation and after 7 days (T7d) and 30 days (T30d) of treatment in the corresponding group. First, in the QLF baseline images (sound enamel) the region of interest adjacent to the orthodontic bracket was defined. This area was recalled on the following images after demineralisation and at T7d and T30d. Average percentage of fluorescence loss with respect to the fluorescence of sound tissue (ΔF, %) and fluorescence loss times the area (ΔQ, % × mm^2^) was calculated by the software.

All measurements were performed blinded to the group allocation. For this, the samples were removed from their container by an individual who was not involved in the study procedure. The same person replaced the samples after measurements for further storage.

All performed methods were carried out in accordance with the relevant guidelines and regulations.

### Statistical evaluation

The data were analyzed using the statistical software MedCalc (v19.2.1). Data were tested for normal distribution using the Shapiro–Wilk’s test to check for normality (p < 0.05). Non-parametric tests (Kruskal–Wallis test and Friedman test) were used for further analysis. The significance level was set at α = 0.05.

## Data Availability

The dataset generated and analysed during the current study was submitted with the manuscript as “Manuscript related file”. After publication the dataset are available from the corresponding author on reasonable request.
